# Left bundle branch area pacing improving the left atrial outcomes in pace‐dependent patients compared with right ventricular outflow tract septal pacing

**DOI:** 10.1002/clc.24185

**Published:** 2023-11-17

**Authors:** Yanlei Zhao, Qian Liu, Jinglan Wu, Yan Zhang, Ling You, Ruiqin Xie

**Affiliations:** ^1^ Department of Cardiology Second Hospital of Hebei Medical University Hebei China

**Keywords:** left atrial strain, left bundle branch area pacing, right ventricular outflow tract septal pacing

## Abstract

**Background:**

Recent studies suggested that the left bundle branch area pacing (LBBAP) has a better efficacy to reduce QRS duration and produce a lower pacing threshold than the conventional right ventricular outflow tract septal pacing (RVOP), which resulted in a better cardiac function and ventricular synchronization. However, whether the LBBAP has a better efficacy in improving left atrial structure, function in pace‐dependent patients compared with RVOP has not been well studied.

**Objective:**

The purpose of this study was to compare the atrial outcomes of pace‐dependent patients who received LBBAP or RVOP procedures.

**Methods and Results:**

A total of 72 patients (including II° AVB, high AVB, and III° AVB, excluding atrial fibrillation patients with atrioventricular block) consecutively enrolled in this single‐center prospective clinical study and randomly assigned to the RVOP group and the LBBP group with 36 patients. All patients were pace‐dependent. The changes in echocardiogram, speckle‐tracking echocardiography, brain natriuretic peptide (BNP), and 6‐min walking distance were documented and compared between two groups at baseline, 7 days, 1, 3, and 6 months after the implantation. There were no significant differences in baseline characteristics between the two groups. The results of the study were as following: (1) left atrial structure index: Our study indicated that there are no significant differences in left atrial anteroposterior dimension (LAAPD), left atrial superoinferior dimension, and left atrial mediolateral dimension between two groups. While the LAAPD in the LBBAP group was significantly reduced at 6 months after implantation ([38.22 ± 2.17] mm vs. [34.13 ± 1.59] mm, *p* < .05). (2) Left atrial strain index: We observed that the S% was significantly improved in both groups at 3 and 6 months after implantation but more prominent in the LBBAP group at 6 months (36.94 ± 11.67 vs. 25.87 ± 8.93, *p* = .01). SRs, SRe were improved in the RVOP group at 6 months after implantation but was further significantly increased in the LBBAP group. Similarly, the SRa in the LBBAP group was significantly better than the RVOP group after 6 months (−2.11 ± 0.75 vs. −2.51 ± 0.70, *p* = .04). (3) Left atrial ejection index: LAEF% in the LBBAP group was significantly improved compared with the RVOP group (60.02 ± 1.88 vs. 53.65 ± 2.45, *p* = .047) and baseline (60.02 ± 1.88 vs. 49.68 ± 2.75, *p* < .05) at 6 months after the surgery. (4) Left ventricular ejection index: The LVEF% in the LBBAP group was significantly increased than the RVOP group after 6 months (69.14 ± 4.99 vs. 64.60 ± 4.84, *p* = .01) and the BNP level was significantly lower in the LBBAP group compared with the RVOP group at 7 days, 1, 3, and 6 months after implantation (*p* < .05). (5) 6‐min walking distance: the 6‐min walking distance was significantly increased at 3 and 6 months after implantation compared with that before (*p* < .05) in both groups, but was more prominent in LBBAP groups ([483.03 ± 11.02] m vs. [431.09 ± 10.69] m,*p* < .05).

**Conclusion:**

Compared with the traditional RVOP, the LBBAP procedure increased left atrial myocardial stress as well as left atrial ejection in pace‐dependent patients at follow‐up to 6 months.

## Graphical abstract

Echocardiography differences between the RVOP and LBBAP groups. Comparison of atrial strain index between the RVOP and LBBAP groups as assessed by S% and LAEF%. We found that the S% increased significantly at 3 and 6 months after implantation in the LBBAP group while decreasing in the RVOP group (*p* < .05). The LAEF% was significantly higher in the LBBAP group compared to the RVOP group at 6 months after implantation.

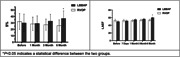

## 1 INTRODUCTION

Permanent pacemaker implantation is an effective method to treat pathological bradycardia and cardiac conduction abnormalities. Long‐term right ventricular apical pacing affects the left atrial (LA) structure and function, which may trigger new‐onset atrial arrhythmias and increase the incidence of atrial fibrillation, heart failure, and pacemaker‐induced cardiomyopathy.^1^, ^2^ Compared with RV apical pacing, although RV septal pacing shows less dyssynchrony and abnormalities in the motion of the LV wall, Kat^3^ also confirmed that it may induce AF in up to one quarter of patients paced for atrioventricular block according to the frequency of pacing. AF induced by RV septal pacing and a paced QRS duration ≥155 ms at pacemaker implantation are significantly associated with poor prognosis. To minimize the risk of such side effects, his bundle area pacing can synchronously activate the left and right ventricles which render it an ideal pacing site. Many studies^4^, ^5^, ^6^, ^7^, ^8^ have indicated that LBBAP is feasible with high success rates and low complication rates during long‐term follow. Studies have indicated^4^, ^5^, ^8^ that the LBBAP can reduce QRS duration, ameliorate electromechanical nonsynchrony, and significantly improve cardiac function as well as ventricular synchronization. Wu et al.^6^ found that the LBBAP has the potential to reduce the risk of pacemaker‐induced cardiomyopathy. Huang^7^ observed similar improvements in LV function with LBBP and HBP, which were significant in patients treated with BVP. Shan et al.^9^ reported on 11 patients with PICM, after the upgrade to permanent HBP, that average LVEF improved, and LVEDD decreased. Meanwhile, there are a few reports about the effect of left bundle branch area pacing (LBBAP) on LA function. Gia^8^ studied that His bundle area pacing compared with RVA pacing resulted in a more physiological LV electromechanical activation and consequently better LA function. The previous study in our center has confirmed^10^ that compared to the right ventricular outflow tract septal pacing (RVOP) method, the QRS interval was significantly shorter in patients who received the LBBAP procedure along with a lower BNP level and increased early left ventricular diastolic filling volume. However, in patients with ventricular pacing dependence, the effect of LBBAP on the structure and function of the LA remains unclear. The purpose of this study was to compare the effects of LBBAP and RVOP methods on the structure and function of LA in pace‐dependent patients.

## 2 METHODS

This study was conducted at the Second Hospital of Hebei Medical University. The protocol of this prospective, controlled trial was approved by the ethics committee of the Second Hospital of Hebei Medical University (approval no. 2022‐1704). A total of 72 patients with pacemaker implantation indications were consecutive prospectively enrolled. Once enrolled, a consecutively numbered envelope was unsealed by a research assistant. The sealed numbers were generated by a computerized random number generator. Based on this number, the patient was random at 1:1 rato assigned to the RVOP group and the LBBAP group. Thirty‐six patients underwent LBBAP (the LBBAP group), and 36 patients underwent RVOP (the RVOP group) from August 2019 to February 2022 after signing the consent form.

The PWR package in R language was used to analyze the sample size required in the study; the results showed that each group needed at least 26 samples.

### 2.1 Research subjects

**Figure 1:**
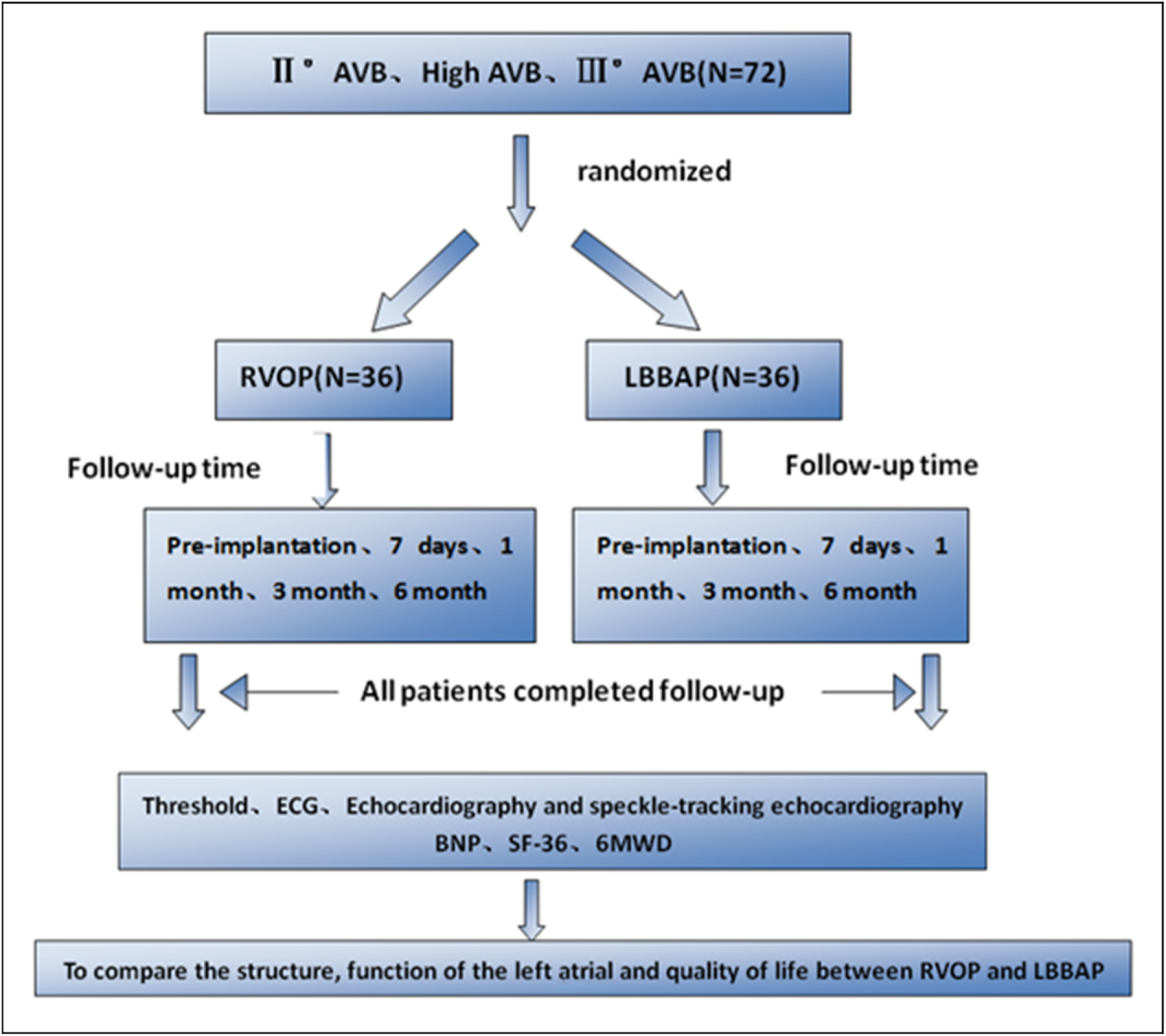
Flowchart of our study. 6MWD, 6‐min walking distance; BNP, brain natriuretic peptide; ECG, electrocardiograph; LBBAP, left bundle branch pacing; RVOP, right ventricular outflow tract septal pacing.

The flowchart of the study is shown in Figure 1. The causes for pacemaker implantation among enrolled patients included: (1) II° atrioventricular block, high atrioventricular block, III° atrioventricular block. (2) Patients with symptoms related to bradyarrhythmias who have consented to a pacemaker installation. The exclusion criteria were as follows: (1) atrial fibrillation patients with atrioventricular block. (2) Patients with atrial septal defect, ventricular septal defect, and other congenital heart disease, rheumatic heart disease, or valvular heart disease. (3) Patients with life expectancy <1 year, complicated with malignant tumors and other related diseases.

### 2.2 Implantation procedures

LBBAP was achieved by applying the transinterventricular septum method in the basal ventricular septum through the 3830 Select pacing leads (Medtronic Inc.) delivered via a fixed sheath (7 F C315 HIS, Medtronic Inc.). During implantation, a unipolar configuration was used for pacing and recording. The delivery sheath was inserted through the left subclavian vein into the atrial side of the tricuspid valve to mark the His bundle potential under fluoroscopic guidance under the right anterior oblique (20°) view. As a marker in the His bundle region, the sheath with the lead tip was further advanced toward the right side of the ventricular septum, approximately 1.5–2 cm, till paced QRS morphology showed left bundle branch block (LBBB) at an output of 2 V/0.42 ms. When the sheath with the lead tip skewed to the left side of the septum, the paced QRS morphology changed from LBBB to right bundle branch block (RBBB) with a gradual change in the notch morphology (“W” waveform) in lead V1 which eventually disappeared. LBB capture was confirmed by the following parameters^5^: (a) paced QRS wave resembles RBBB pattern; (b) left bundle potential could be recorded; (c) pacing stimulus to left ventricular activation time (Sti‐LVAT) shortened abruptly with increasing output or stabilized at both low and high outputs; and (d) selective LBBAP The capture threshold and pacing impedance were routinely measured and recorded during the procedure. R‐wave amplitudes were also constantly measured and maintained at a level higher than 5 mV. Another active electrode was implanted in the right auricle (Supporting Information: Figure 1).

The RVOP procedure was conducted conventionally. The right ventricular pacing lead was positioned in the low intervals of the right ventricular outflow tract, and an active atrial lead was positioned at the right auricle.

### 2.3 Pacemaker programming

All pacemakers were programmed by the same specialist. Pacing thresholds and impedances were recorded at baseline, 1 month, 3 months, and 6 months after implantation.

### 2.4 BNP examination

The BNP levels of all enrolled patients were measured at baseline, 7 days, 1 month, 3 months, and 6 months of pacemaker implantation.

### 2.5 Electrocardiograph

All patients received routine electrocardiogram examinations throughout the study. The following parameters were documented at baseline, 1 month, 3 months, and 6 months: QRS duration, QRS amplitude, QRS duration, and morphology were carefully measured intraoperatively. This examination was repeated by two cardiologists independently and the results were averaged.

### 2.6 Echocardiography and speckle‐tracking echocardiography

All patients received conventional transthoracic echocardiography (iE33 machines equipped with X3; Philips Medical Systems) at baseline and 1 month, 3 months, and 6 months after implantation. The following indexes were measured: (1) LA structure index: left atrial anteroposterior dimension (LAAPD); left atrial mediolateral dimension (LAMD); left atrial superoinferior dimension (LASID); (2) LA strain index: LA strain and strain rate were measured by speckle‐tracking echocardiography (STE). We manually monitored three markers under the LA maximum volume view, acquired the curve of the strain rate (SR‐LAs, SR‐LAe, SR‐LAa), and calculated the average value under both the two‐chamber viewers.^11^ (3) LAVmax, LAVmin, and Left atrial ejection index (LAEF%): Total atrial emptying fraction = ([LAVmax−LAVmin]/[LAVmax] × 100%. (4) Left ventricular function index:early diastolic velocity at the septal mitral annulus (e′); peak E‐wave velocity (E); E/e′; left ventricular ejection fraction (LVEF%); and velocity‐time integration of aortic blood flow (VTI). The images were recorded over three consecutive cardiac cycles with a stable echocardiographic appearance (Supporting Information: Figure 2).

### 2.7 Statistical methods

All data were analyzed by SPSS Statistics software version 23.0. The K–S test was applied to verify whether the data followed a normal distribution. Descriptive statistics were used for continuous variables, which were presented as the mean and standard deviation. Student *t* test was used to compare means, and the *X* ^2^ test was used for qualitative data. Repeated measurements were compared using analysis of variance. A *p* value < .05 was considered statistically significant. The graphs in this article were drawn using GraphPrism.

## 3 RESULTS

### 3.1 Baseline characteristics

A total of 72 patients were consecutively enrolled in this study from August 2019 to February 2022, including 36 patients (mean age of 68.11 ± 10.04 years; 18 males) in the RVOP group and 36 patients (mean age of 64.26 ± 14.10 years; 20 males) in the LBBAP group. All procedures were successfully carried out in both groups. As presented in Table 1, no significant difference in age, gender, or medical histories, including hypertension, diabetes, heart failure, cerebrovascular disease, was identified between the two groups. Among patients who received the LBBAP procedure, 4 were diagnosed with II°II AVB, 5 with high ABV, and 27 with III°AVB. In the RVOP group, 5 patients were diagnosed with II°AVB, 6 with high AVB, and 25 patients with III°AVB. No procedure‐associated adverse events were observed during the study.

**Table 1:** Comparison of baseline characteristics between the RVOP group and the LBBAP group.

Baseline	RVOP (*n* = 36)	LBBAP (*n* = 36)	*p*
Age (years)	68.11 ± 10.04	64.26 ± 14.10	.352
Male sex	18 (50.00%)	20 (55.56%)	.637
Hypertension	21 (58.34%)	25 (69.44%)	.326
Diabetes	7 (16.67%)	9 (21.43%)	.571
Heart failure	16 (44.45%)	15 (41.67%)	.812
Cerebrovascular disease	14 (38.89%)	14 (38.89%)	1
Hyperlipidemia	7 (19.44%)	6 (16.67%)	.759
RBBB	2 (4.76%)	6 (14.29%)	.134
LBBB	0 (0.00%)	1 (2.78%)	.314
II°AVB	5 (13.89%)	4 (11.11%)	.722
High AVB	6 (16.67%)	5 (13.89%)	.743
III°AVB	25 (69.44%)	27 (75.00%)	.793

*Note*: Data were presented as mean ± standard deviation (SD) for continuous variables or number of subjects (*n*) and percentage (%) for categorical variables.

Abbreviations: AV, atrioventricular block; LBBB, left bundle branch block; LBBAP, left bundle branch area pacing; RBBB, right bundle branch block; RVOP, right ventricular outflow tract septal pa.

### 3.2 Pacing threshold and impedance and ECG of the left bundle branch pacing and RVOP

The differences in pacing threshold and impedance between the two groups were summarized in Table 2. Our data suggested that there was no significant difference between the intraoperative pacing threshold of the LBBAP group and the RVOP group ([0.99 ± 0.56] V vs. [0.92 ± 0.19],*p* = .212). Similarly, there was no significant difference in thresholds between the two groups at 6 months after operation. At 6 months after implantation, the threshold significantly decreased in both groups compared to baseline (*p* < .05). The average intraoperative impedance in the LBBAP group was significantly higher than that of the RVOP group ([772.7 ± 116.12] Ω vs. [751.48 ± 67.240 Ω *p* = .023]; At 6 months after the operation, the impedance of the LBBAP group remained significantly lower than that of the RVOP group[(538.18 ± 92.62) Ω vs. (580.62 ± 87.90) Ω *p* = .032].

**Table 2:** Comparison of pacing threshold, impedance, and ECG parameters between the RVOP and the LBBAP groups.

		RVOP	LBBAP	*p* for group
Threshold (V)	At implantation	0.92 ± 0.04	0.99 ± 0.15	.212
	6‐month follow‐up	0.66 ± 0.61	0.78 ± 0.66	.234
	*p* for time	.025	.012	
Impedance (Ω)	At implantation	751.48 ± 67.24	772.7 ± 116.12	.023
	6‐month follow‐up	580.62 ± 87.90	538.18 ± 92.62	.032
	*p* for time	<.001	<.001	
QRS duration (ms)	At implatation	122.21 ± 18.94	124.31 ± 17.82	.603
	6‐month follow‐up	153.43 ± 12.30	104.66 ± 12.07	<.001
	*p* for time	<.001	<.001	
QRS Amplitude (mV)	At implatation	2.42 ± 0.91	2.14 ± 0.69	.062
	6‐month follow‐up	2.41 ± 1.52	2.28 ± 0.79	.244
	*p* for time	.30	.29	

*Note*: Values are mean ± (SD).

Our data indicated that the average duration of the QRS wave was significantly prolonged at 6 months after surgery in the RVOP group ([122.21 ± 18.94] ms vs. [153.43 ± 12.30] ms,*p* < .001) and significantly reduced in the LBBB group compared to baseline [124.31 ± 17.82] ms vs. [104.66 ± 12.07] ms,*p* < .001). On the other hand, our results suggested that the average QRS width of the LBBAP group was significantly shorter than that of the RVOP group at the same time point ([153.43 ± 12.30] ms vs. [104.66 ± 12.07] ms,*p* < .001). Meanwhile, no significant difference in the QRS amplitude was identified both between and within groups (*p* > .05) (Table 2).

### 3.3 Echocardiography differences between the two groups

**Figure 2:**
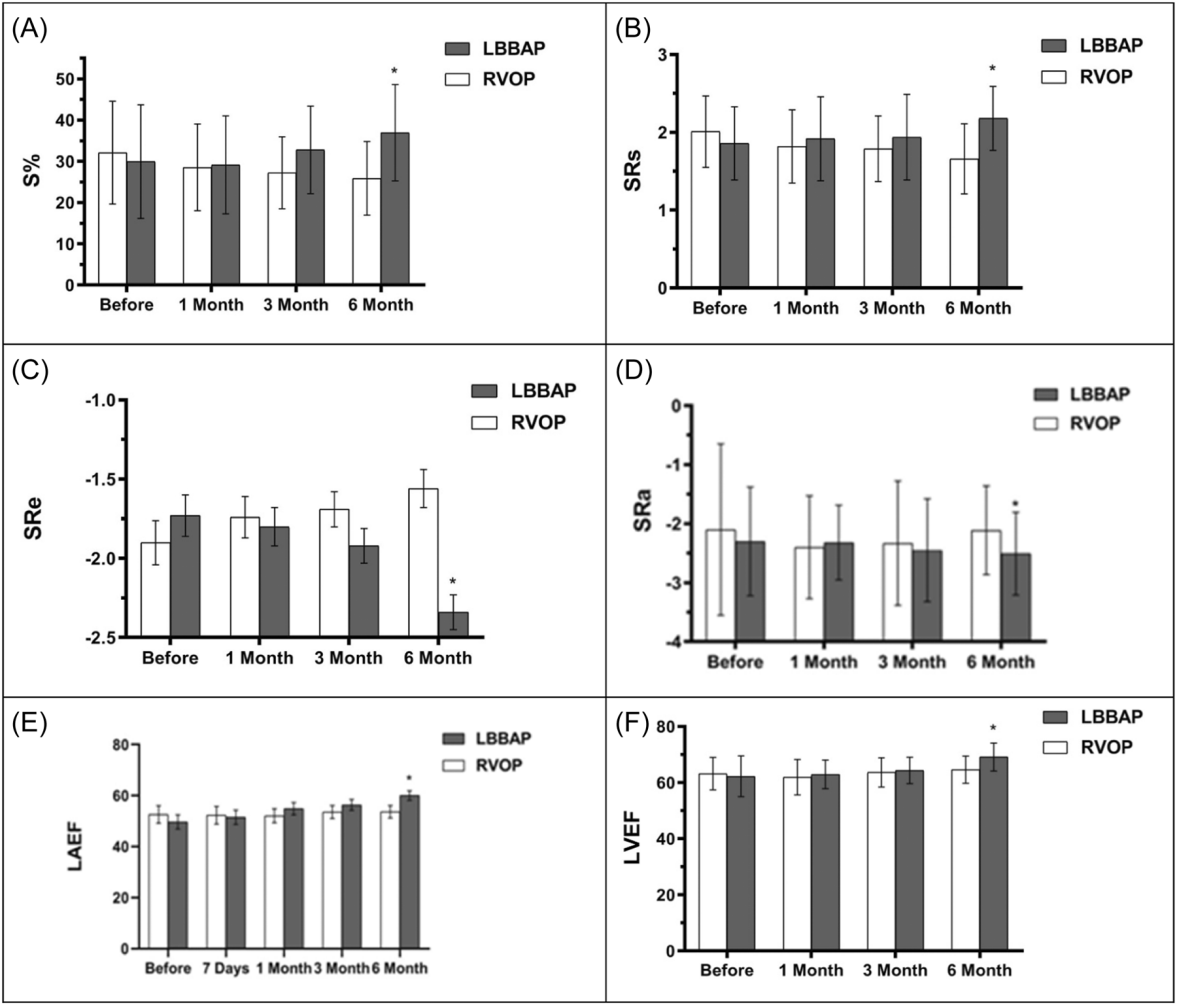
(A) Comparison of atrial strain index between the two groups as assessed by S%, SRe, SRs, and SRa. (a–d). We found that the S% increased significantly at 3 and 6 months after implantation in the LBBAP group while decreased in the RVOP group (*p* < .05). The SRs, SRe, and SRa value was significantly increased in the LBBAP group compared to the RVOP group at 6 months after implantation (*p* < .05). (B) Comparison of atrial function, as assessed by LAEF% between the two groups. We found that the LAEF% was significantly higher in the LBBAP group compared to the RVOP group at 6 months after implantation. (C) Comparison of left ventricular function, as assessed by LVEF% between the two groups. Our data suggested that the LVEF% In the LBBAP group was significantly higher than that of RVOP groups at 3 and 6 months after implantation. Variables are expressed as means±standard deviations. *p < .05 indicates a statistical difference between the two groups. LBBAP, left bundle branch area pacing; LAEI; Left atrial ejection index; RVOP, right ventricular outflow tract septal pacing.

A comparison of echocardiographic parameters between the two groups was presented in Table 3. (1) Left atrial structure index: The LAAPD decreased significantly at 6 months after implantation in the LBBAP group ([38.22 ± 2.17] mm vs. [34.13 ± 1.59] mm,*p* < .05), but no significant difference in the LAAPD, LAMD, and LASID was identified between the two groups. (2) Left atrial strain index: The S% increased significantly at 3 and 6 months after implantation in the LBBAP group while decreasing in the RVOP group (*p* < .05). The SRs, SRe, and SRa value was significantly increased in the LBBAP group compared to the RVOP group at 6 months after implantation (*p* < .05).(3) Left atrial ejection: The LAVmin was significantly higher in the RVOP group compared to the LBBAP group at 6 months after implantation (35.42 ± 17.46 vs. 31.76 ± 16.15,*p* = .02). The LAEF% was significantly higher in the LBBAP group compared to the RVOP group at 6 months after implantation (60.02 ± 1.88 vs. 53.65 ± 2.45,*p* = .04). (4) Left ventricular function index: The mean E/e′ ratio of the LBBAP group was significantly lower than that of the RVOP group at 6 months after implantation (10.63 ± 2.37 vs. 12.35 ± 1.29, *p* = .02). In addition, our data suggested that the LVEF% In the LBBAP group was significantly higher than that of RVOP groups at 3 and 6 months after implantation (63.63 ± 5.20 vs. 64.32 ± 4.73 *p* = .02, 64.60 ± 4.84 vs. 69.14 ± 4.99 *p* = .01) (Figure 2, Table 3).

**Table 3:** Comparison of echocardiographic parameters between the RVOP and LBBAP groups.

		RVOP	LBBAP	*P*
LAAPD	Pre	34.41 ± 1.97	38.22 ± 2.17	.212
	7d‐after	32.57 ± 1.46	35.86 ± 1.43	.186
	1m‐after	34.85 ± 1.63	37.32 ± 1.58	.176
	3m‐after	33.83 ± 1.74	35.71 ± 1.61	.455
	6m‐after	34.88 ± 2.87	34.13 ± 1.59^a^	.782
LASID	Pre	44.23 ± 4.95	51.50 ± 3.52	.433
	7d‐after	48.91 ± 3.53	54.92 ± 2.52	.425
	1m‐after	49.00 ± 4.24	50.21 ± 3.28	.879
	3m‐after	55.36 ± 0.70	48.50 ± 0.5	.084
	6m‐after	51.28 ± 4.25	49.33 ± 0.32	.879
LAMD	Pre	31.67 ± 2.31	37.33 ± 4.62	.133
	7d‐after	38.67 ± 3.21	34.67 ± 0.58	.104
	1m‐after	39.12 ± 4.36	34.23 ± 1.28	.129
	3m‐after	37.34 ± 4.58	33.67 ± 2.31	.323
	6m‐after	36.67 ± 5.56	32.33 ± 0.57	.254
E/e′	Pre	14.59 ± 1.23	12.83 ± 1.97	.343
	7d‐after	13.20 ± 1.29	10.83 ± 1.96	136
	1m‐after	14.04 ± 6.92	11.07 ± 1.28	.354
	3m‐after	12.53 ± 2.15	11.15 ± 1.95	.172
	6m‐after	12.35 ± 1.29	10.63 ± 2.37	.025
LAVmax	Pre	60.62 ± 15.24	58.98 ± 18.76	.201
	7d‐after	60.04 ± 14.12	58.05 ± 13.24	.241
	1m‐after	62.04 ± 20.55	58.17 ± 21.29	.143
	3m‐after	61.38 ± 14.40	57.18 ± 17.39	.430
	6m‐after	61.16 ± 15.56	58.68 ± 18.79	.256
LAVmin	Pre	33.02 ± 12.19	32.76 ± 11.65	.568
	7d‐after	33.04 ± 14.12	33.08 ± 11.12	.457
	1m‐after	34.90 ± 19.04	32.52 ± 13.14	.046
	3m‐after	35.38 ± 9.66^a^	32.18 ± 11.74	.032
	6m‐after	35.42 ± 17.46^a^	31.76 ± 16.15	.025
LAEF%	Pre	52.61 ± 3.45	49.68 ± 2.75	.482
	7d‐after	52.27 ± 3.47	51.50. ± 2.78	.868
	1m‐after	52.10 ± 2.76	54.84 ± 2.43	.446
	3m‐after	53.55 ± 2.54	56.36 ± 2.13	.395
	6m‐after	53.65 ± 2.45	60.02 ± 1.88^a^	.047
LVEF%	Pre	63.18 ± 5.80	62.27 ± 7.23	.517
	1m‐after	61.93 ± 6.32	62.93 ± 5.12	.700
	3m‐after	63.63 ± 5.20	64.32 ± 4.73^a^	.587
	6m‐after	64.60 ± 4.84	69.14 ± 4.99^a^	.046

^a^ Represent for compared with pre‐implantation, *p* < .05.

### 3.4 BNP and 6‐min walking distance

As presented in Table 3, the BNP level was significantly decreased in both groups after implantation compared to baseline, but more prominent in the LBBAP group compared to the RVOP group at 7 days ([137.19 ± 12.31] pg/mL vs. [06.74 ± 25.93] pg/mL, *p* < .01), 1 month ([95.28 ± 8.89] pg/mL vs. (73.20 ± 10.87) pg/mL, *p* < .01), 3 months ([71.90 ± 5.04] pg/mL vs. [27.76 ± 4.45] pg/mL, *p* < .01). The difference between the two groups was most obvious 6 months after implantation ([56.17 ± 4.13] pg/mL vs. [14.16 ± 3.64] *p* < .01). The 6‐min walking distance was significantly increased at 3 months and 6 months after implantation in both groups compared to baseline and there was statistical significance identified between the two groups ([431.09 ± 10.69] m vs. [483.03 ± 11.02] m,*p* = .001).

## 4 DISCUSSION

Right ventricular apex (RVA) pacing has adverse effects on left atrial (LA) function and may contribute to atrial arrhythmias.^12^ The RV septum represents another selective pacing site which results in a more physiological LV electromechanical activation than RVA pacing.^13^ But Hilock has suggested that the right ventricular outflow tract septum might affect LV function after 12–18 months.^14^ Kat also confirmed that the percentage of RV pacing contributes significantly to the development of AF in patients with AV block. The LBBAP was first proposed by Huang^15^ in 2017 and quickly became rather popular in the field since it can enable the synchronization of left ventricular contraction and improve postoperation cardiac function. Although the LBBAP has good electrical parameters and stabilization,^11^, ^15^ the effects of left bundle area pacing in pace‐dependent patients on LA structure and function are still lacking. In this study, we depicted and compared the LA structure and strain, BNP, and SF‐36 of LBBAP and RVOP at baseline as well as 7 days, 1 month, 3 months, and 6 months after implantation.

### 4.1 Pacing threshold and impedance

LBBAP can cross the block site which leads to a relatively low and stable low acquisition threshold. Consistent with Chen et al.^16^, ^17^, ^18^ found in their studies that the seizure threshold was usually <1 V at 0.5 ms, the average pacing threshold of the LBBAP group was low and relatively stable at the implantation and 6 months after the implantation ([0.99 ± 0.56] V vs. [80.78 ± 0.20] V *p* < .001). This is similar to our results. The low and stable acquisition threshold avoids the situation of cross‐perception of the atrial Hirschner beam wire. Impedance of both LBBAP and RVOP was decreased at 6 months postoperatively compared to baseline.

### 4.2 QRS width of ECG

The duration of QRS wave has been widely used in clinics to estimate cardiac electrical synchronization conditions. Pang et al.^19^ found that prolonged QRS duration not only affected left ventricular systolic function, but also increased the risk of heart failure by reducing left ventricular diastolic function.^17^ Recent studies suggested that prolonged QRS duration is a risk factor for developing heart failure in patients with LBBB.^19^ Li et al.^17^ demonstrated in their study that the QRS duration in patients who received LBBAP treatment was shorter than that of RVSP ([113.2 ± 9.9] ms vs. [144.4 ± 12.8] ms, *p* < .001) which was consistent with Shigeng's study^20^ ([111.83 ± 6.89] vs. [155.36 ± 5.94], *p* = .000]. In our study, the average duration of QRS wave in the LBBAP group was decreased compared to baseline ([124.31 ± 17.82] ms vs. [104.66 ± 12.07] ms, *p* < .001]. While the postoperative QRS duration in the RVOP group was significantly prolonged indicating that the LBBAP maintained a better ventricular electrical synchronization than the RVOP.

### 4.3 Left atrial structure and function

Left atrium stores pulmonary venous return blood and participates in the diastole phase through different mechanisms. Left atrial function includes three phases: (a) reservoir (inflow during ventricular systole), (b) conduit (passive emptying during ventricular relaxation and diastasis), and (c) contraction (active emptying near ventricular end‐diastole). In this study, left atrial function was evaluated in all patients by measuring the strain rate peaks (SRs, SRe, and Sra) of each wall of the left atrium during systolic, early diastolic, and late diastolic periods. Inaba^21^ found that on the left atrial strain rate curve, SRs (positive wave) indicates the function of the reservoir, SRe (negative wave) for the function of pipeline, and SRa (negative wave) reflects the function of auxiliary pump. In this study, We observed that the S%, SRs, SRe, and SRa in LBBAP group were significantly improved in 6 months after implantation compared with RVOP. Previous studies have indicated that with longer periods of pacing, the underlying ventricular dysfunction contributes to LA remodeling/stiffness, further decreasing the LA function.^22^ Kat confirmed that cumul%VP was an independent predictor of postimplantation AF in patients with no history of AF.^3^ The reservoir and conduit phases of the LA function are related to the common mechanism of RV pacing‐induced systolic and diastolic dysfunction.^22^ Our studies indicated that LBBAP, as a more physiological pacing method, can achieve atrioventricular synchronization while preserving left atrial function. E/e′ is the ratio of the left ventricular early diastolic filling velocity on the mitral orifice flow velocity curve to the mitral annulus early diastolic flow velocity. E/e′ is highly correlated with left atrial pressure which is a risk factor for the occurrence of atrial fibrillation and heart failure as well as pacemaker‐associated cardiomyopathy in patients who received pacemaker implantation. In this study, the E/e ratio in the LBBAP group was significantly higher than that in the RVOP group, indicating that LBBAP could improve ventricular diastolic function and reduce left atrial pressure. Many studies^23^, ^24^ have demonstrated that right ventricular outflow pacing increased the risk of atrial fibrillation and heart failure due to left ventricular desynchrony which increases mitral regurgitation and left atrial pressure. In this study, the LAVmin was significantly higher in the RVOP group compared to the LBBAP group at 6 months after implantation. This result shows that LBBAP is a physiological form of pacing. The increase in left ventricular end‐diastolic pressure was less than that in the RVOP group. As a result, it causes a smaller LAVmin increase. The LAEF% in the LBBAP group significantly increased at 6 months after implantation compared to the RVOP group. In summary, our data indicated that the LBBAP increased left atrial stress, reduced left atrial pressure, and improved left atrial ejection. Compared to the RVOP, the LBBAP not only has a better efficacy in improving cardiac synchronization but also perseveres and might even improve the left atrial function.

### 4.4 Left ventricular function

Many previous studies^20^, ^25^, ^26^ have shown that LBBAP can maintain cardiac function stability and reduce the risk of heart failure compared with right ventricular pacing. Shigeng^20^ which enrolled patients with nonischemic cardiomyopathy who had successfully received LBBAP, indicated that LVEF% could be effectively improved 1 year after surgery in patients with reduced end‐systolic volume of the left ventricle (33 ± 8% vs. 55 ± 10%, *p* < .001) and The NYHA functional classification increased from 2.8 ± 0.6 to 1.4 ± 0.6. In our studies, the findings are comparable to the published studies by other authors on LBBAP.

### 4.5 BNP and 6WMD

BNP, as a quantitative indicator of heart failure, can reflect the systolic and diastolic functions of the left ventricle.^22^, ^25^, ^26^ Similar case reports^27^, ^28^ also showed that in patients with heart failure who received LBBAP, their NYHA function grade improved along with cardiac parameters. Shunmuga et al.^29^ found that in patients older than 80, LBBAP can be used as an effective alternative therapy for CRT to improve cardiac function. Margarida^30^ reported that LBBAP could correct the cardiac electromechanical dyssynchronization and ameliorate mitral regurgitation. In this study, the BNP level was significantly decreased in both groups compared to baseline but more prominent in the LBBAP group compared to the RVOP group at all time points, indicating that although RVOP could achieve some certain levels of atrioventricular sequential contraction, it was still far inferior to physiological pacing. 6WMD is widely used in clinical practice endurance evaluation for patients with heart disease. It is an important index to evaluate the treatment outcomes in patients with chronic heart failure.^22^ The 6WMD in the LBBAP group was significantly improved compared to the RVOP group at 3–6 months after surgery, indicating that LBBP can effectively increase quality of life and exercise tolerance in pace‐dependent patients.

## 5 CONCLUSION

Compared with the traditional RVOP, the LBBAP procedure not only improved the left ventricular ejection and 6‐min walking distance, but also increased left atrial myocardial stress as well as left atrial ejection in pace‐dependent patients at follow‐up to 6 months.

## 6 LIMITATIONS AND SHORTCOMINGS

(1) The number of patients enrolled in this single‐center study was relatively small and might not be representative of the population. In addition, the follow‐up time was relatively short to observe any potential long‐term side effects of this novel technique.
(2) Due to the limited number of patients, this study was not powered to identify the differences based on different etiologies for pacemaker implantation. Future studies, including more subjects and more comprehensive design, should be conducted to better understand the merit of this novel technique.

## CONFLICT OF INTEREST STATEMENT

The authors declare no conflict of interest.

## Supporting information

Supporting information.

## Data Availability

Due to the nature of this research, participants of this study did not agree for their data to be shared publicly, so supporting data are not available.
