# Supplementary material for: Left bundle branch area pacing improving the left atrial outcomes in pace‐dependent patients compared with right ventricular outflow tract septal pacing
Source: Clin Cardiol. 2023 Nov 17;47(2):e24185. doi: 10.1002/clc.24185 (PMC10823449; doi:10.1002/clc.24185)
Supplement: Supplementary file 1 — Supporting information. [file CLC-47-e24185-s001.docx]

| **Supplementary figure 1** | | | | |
| --- | --- | --- | --- | --- |
| 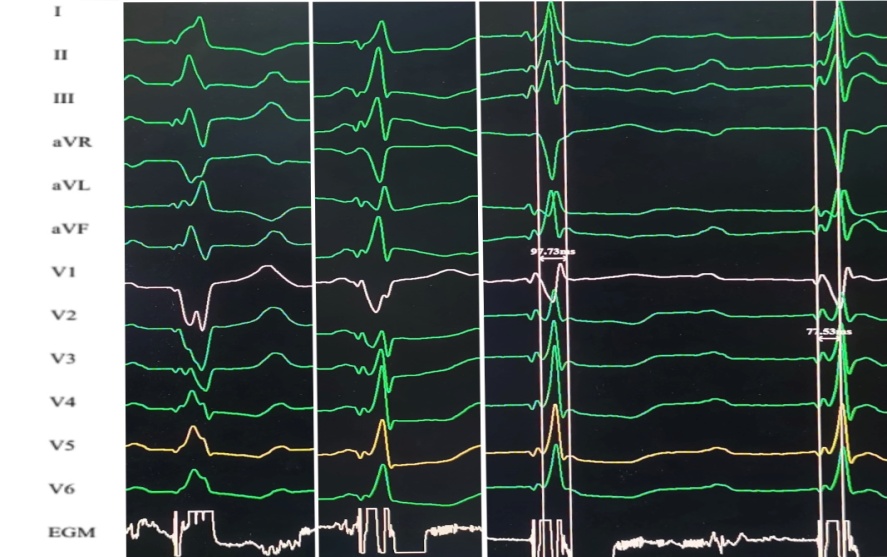 | | | | |
|  | **A** | **B** | **C** |  |
| Supplementary Fig. 1.Twelve‑leads ECG and intracardiac electrocardiogram(EGM) during LBBAP in a patient:(A). Paced morphology of “W” pattern with a notchatthe nadir of the QRS and gradually ascend in lead V1.(B-C) With increased output from 2 V/0.42 ms to 5 V/0.42 ms, the stimulus to left ventricular activation time remains constant (77.53 ms) with impedance of 750 Ω and the QRS duration was 97.73ms. | | | | |

| **Supplementary figure 2** | |
| --- | --- |
| 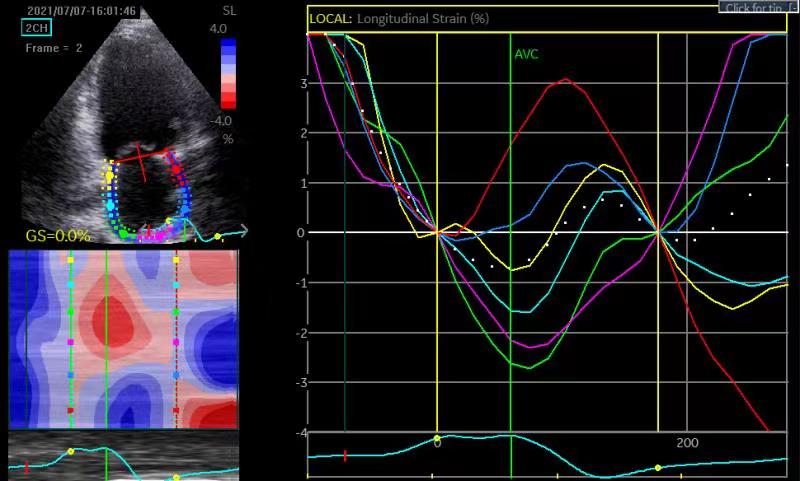 | 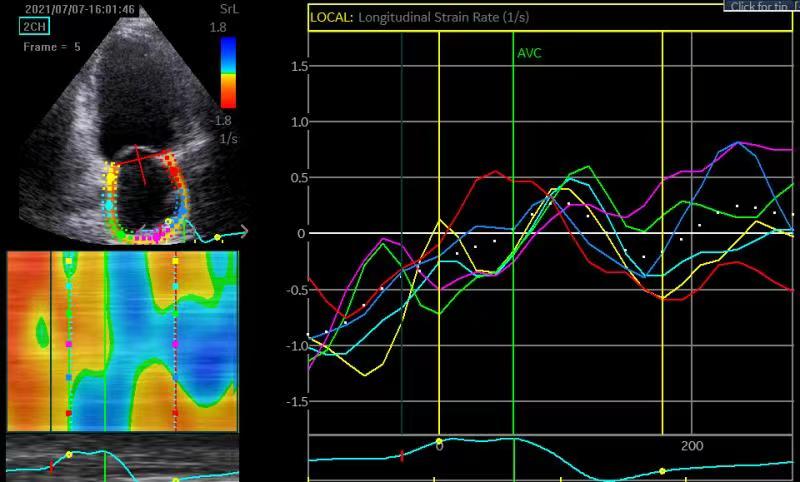 |
| **a** | **b** |
| Supplementary Fig.2 Left atrial strain index measured by speckle tracking echocardiography.(a.b).Measurement of LA strain (S%)and strain rate(SRs,SRa,SRe) in apical two-chamber view. | |
